# Identification of miRNA-Mediated Core Gene Module for Glioma Patient Prediction by Integrating High-Throughput miRNA, mRNA Expression and Pathway Structure

**DOI:** 10.1371/journal.pone.0096908

**Published:** 2014-05-08

**Authors:** Chunlong Zhang, Chunquan Li, Jing Li, Junwei Han, Desi Shang, Yunpeng Zhang, Wei Zhang, Qianlan Yao, Lei Han, Yanjun Xu, Wei Yan, Zhaoshi Bao, Gan You, Tao Jiang, Chunsheng Kang, Xia Li

**Affiliations:** 1 College of Bioinformatics Science and Technology, Harbin Medical University, Harbin, China; 2 Department of Neurosurgery, Tiantan Hospital, Capital Medical University, Beijing, China; 3 Department of Neurosurgery, Tianjin Medical University General Hospital, Laboratory of Neuro-Oncology, Tianjin Neurological Institute, Laboratory of Neurotrauma, Variation and Regeneration, Ministry of Education and Tianjin Municipal Government, Tianjin, China; 4 Department of Bioinformatics, School of Basic Medical Sciences, Fujian Medical University, Fuzhou, China; Swedish Medical Center, United States of America

## Abstract

The prognosis of glioma patients is usually poor, especially in patients with glioblastoma (World Health Organization (WHO) grade IV). The regulatory functions of microRNA (miRNA) on genes have important implications in glioma cell survival. However, there are not many studies that have investigated glioma survival by integrating miRNAs and genes while also considering pathway structure. In this study, we performed sample-matched miRNA and mRNA expression profilings to systematically analyze glioma patient survival. During this analytical process, we developed pathway-based random walk to identify a glioma core miRNA-gene module, simultaneously considering pathway structure information and multi-level involvement of miRNAs and genes. The core miRNA-gene module we identified was comprised of four apparent sub-modules; all four sub-modules displayed a significant correlation with patient survival in the testing set (P-values≤0.001). Notably, one sub-module that consisted of 6 miRNAs and 26 genes also correlated with survival time in the high-grade subgroup (WHO grade III and IV), P-value = 0.0062. Furthermore, the 26-gene expression signature from this sub-module had robust predictive power in four independent, publicly available glioma datasets. Our findings suggested that the expression signatures, which were identified by integration of miRNA and gene level, were closely associated with overall survival among the glioma patients with various grades.

## Introduction

Glioma is the most common form of primary brain tumor, accounting for 7% of the years of life lost from cancer before the age of 70 [1], [2]. According to the World Health Organization (WHO) criteria, glioma tumors are histologically separated into Grade I through IV. Despite significant improvements in treatments for glioma patients, the median survival remains poor, particularly for those with glioblastoma (GBM, grade IV). Patients with newly diagnosed GBM exhibit a median survival of approximately one year, with generally poor responses to all therapeutic modalities [3]. Thus, elucidation of the glioma survival event is important and could potentially aid in the diagnosis and prognosis of glioma patients.

MicroRNAs (miRNAs) are a class of non-coding RNAs able to regulate gene expression at the post-transcriptional level by binding to the 3′ untranslated region of target messenger RNAs (mRNAs) and causing a block of translation and/or mRNA degradation [4]. Recently, a growing level of attention has been focused on the biological interplay between mRNA expression in conjunction with corresponding miRNA data in various cancer types, including glioma [5], [6], [7], [8]. Thus, the amount of sample-matched miRNA-gene profiles (miRNA and gene expression profiles quantified using exactly the same set of biological samples) is rapidly increasing for such miRNA-gene integrative analysis. More importantly, the idea that many biological factors are coordinated at the network level rather than an individual molecular level has been accepted [3]. And some studies have interrogated kinds of networks to understand the complex regulatory mechanisms in the glioma, for example the miRNA-TF mediated regulatory network [9]. As a biological network, pathway provides reliable topology structure information which could be a platform for multi-dimensional data integration. Recently, biological pathways have been applied to explore the mechanism involved in many aspects, including disease occurrence, miRNA regulation and drug action [10], [11], [12].

Focusing on glioma survival event, many experimental studies have demonstrated that the regulatory function of miRNAs on genes, which further affects key biological pathways, plays a role in cell survival process. For example, tumor-suppressive miR-326 regulated Notch pathway, an important glioma cell survival pathway, by mediating the toxic effects of *notch* knockdown [13], [14]. MiR-221 and miR-222 induced cell survival in GBM by targeting pro-apoptotic gene *PUMA* in the mitochondrial apoptotic pathway [15]. In the present, some microarray studies have tried to explore glioma cell survival mechanism and identify signature for predicting patient clinical outcome at the gene level [16] or miRNA level [17]. In a systematic perspective, the glioma survival process is also coordinated at the multiple miRNA-gene regulation interactions. However, the systematic integration of miRNA and mRNA expression for analyzing glioma patient survival has not been carefully studied to date. And only a small part of genes as core factors play an important role in glioma patient survival prediction. The integrated analysis of multi-dimensional data has the potential power to identify core and robust survival signatures, which could effectively predict the clinical outcome of glioma patients.

In this study, we have profiled sample-matched miRNA-mRNA expression data from 160 glioma tumors to systematically analyze glioma survival. In the analytical process, we considered the joint impact of miRNAs and genes to identify glioma survival related pathways, and then developed a pathway-based random walk (PbRW) method to identify a glioma core miRNA-gene module. After dissecting the core miRNA-gene module, we verified that one sub-module which consisted of 6 miRNAs and 26 genes displayed a power to predict the clinical outcome of glioma patients.

## Materials and Methods

### Datasets

#### Our dataset and patient information

The sample-matched miRNA and mRNA expression profiling upon 160 glioma samples were collected from the Chinese Glioma Genome Atlas (CGGA, http://www.cgcg.org.cn/) [18], [19]. The 160 glioma cases included 63 WHO grade II patients (50 astrocytomas and 13 oligodendrogliomas), 33 grade III patients (8 anaplastic astrocytomas, 10 anaplastic oligodendrogliomas and 15 anaplastic oligoastrocytomas) and 64 GBM patients (60 primary GBM and 4 secondary GBM). In this study, we identified a glioma core miRNA-gene survival module by integrating analysis of the sample-matched miRNA and mRNA expression data.

#### Gene Expression Omnibus datasets

Four independent mRNA expression datasets with patient survival information were from the following studies: Freije et al. [16], Phillips et al. [20], Murat et al. [21] and Lee et al. [22]. We extracted corresponding raw data from Gene Expression Omnibus (GEO) database [23] (accession number: GSE4412, GSE4271, GSE7696, and GSE13041). In all datasets, we eliminated the glioma samples who had survival time less than 30 days, since these samples might have died for reasons other than the disease itself [17]. Then four expression profilings of 73, 77, 76, and 191 samples were utilized in this study. All expression profilings were created and normalized using RMA algorithm in the Bioconductor affy package (version 1.28.1).

#### The Cancer Genome Atlas datasets

Independent sample-matched miRNA and gene expression datasets were downloaded from TCGA database (http://tcga-data.nci.nih.gov/docs/publications/gbm_exp/). Level three data gave calls for miRNAs and genes per sample after quantile normalization and background correction. The average expression values were calculated for duplicated samples. Only tumor samples were considered in our study. In addition, we eliminated samples with Karnofsky's score less than 70 and survival time less than 30 days, since these patients might have died for reasons other than the disease itself [17]. Finally, a total of 276 patients who fit these criteria and exhibited common miRNA and gene expression were utilized in this study.

#### MiRNA target genes

We acquired miRNA target genes from eleven common miRNA target predicting datasets: DIANA-microT [24], mirSVR [25], PicTar5 [26], RNA22 [27], RNAhybrid [28], TargetScan [29], PITA [30], MirTarget2 [31], TargetMiner [32], miRanda [33], and two valid databases [34], [35]. Firstly, we obtained all miRNA-gene regulation information from these eleven prediction datasets. In order to improve the reliability of the predicted target genes, we extracted only the corresponding target regulations that emerged from at least six of the datasets listed above.

#### Biological pathways information

The information regarding biological pathways was obtained from Kyoto Encyclopedia of Genes and Genomes (KEGG) PATHWAY database [36]. We applied Bioconductor package iSubpathwayMiner [37], [38] to obtain all the biological pathways, including 150 metabolic pathways and 150 non-metabolic pathways. We utilized these pathways to identify glioma survival related pathways.

### Methods

#### The framework

**Figure 1:**
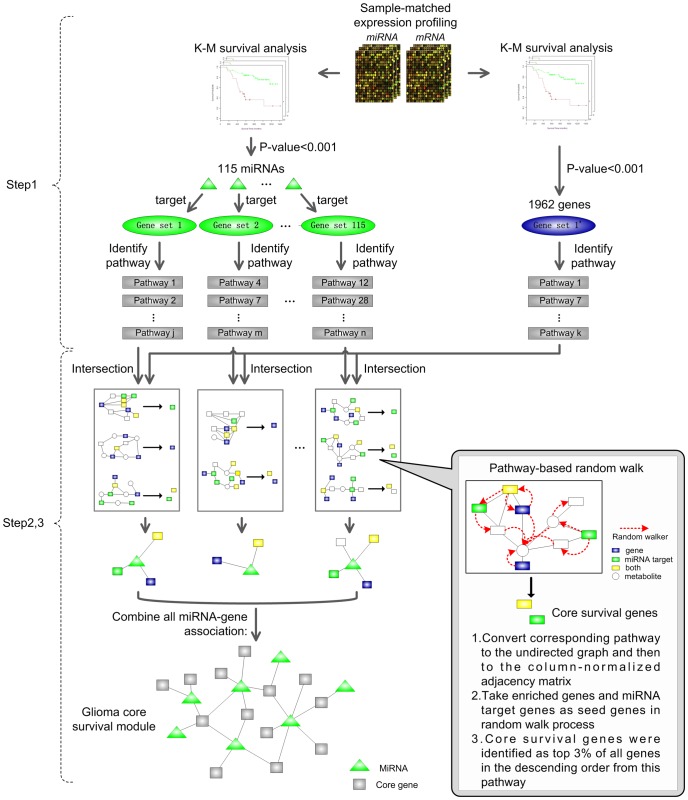
Workflow overview. We identified a glioma core survival module based on sample-matched miRNA, mRNA expression and pathways structure. First, we utilized Kaplan-Meier (K-M) survival analysis to identify glioma survival related miRNAs and genes. Then, we integrated these miRNAs and genes to further identify KEGG pathways. Finally, we developed a pathway-based random walk method to identify glioma core survival genes from each pathway, and constructed a glioma core miRNA-gene survival module.

The 160 glioma cases were randomly divided into a training set (n = 80) and a testing set (n = 80). Table 1 lists the clinicopathological characteristics of patients in both sets, and the entire set. In the following, we performed an integrated analysis of the sample-matched miRNA and mRNA expression data using the training set. As shown in Figure 1, the framework included following steps. In Step1, we used Kaplan-Meier survival analysis to identify glioma survival related miRNAs and genes, and then integrated these miRNAs and genes to further identify glioma survival related pathways. In Step2, we developed pathway-based random walk to identify glioma core survival genes from these pathways based on the pathway structure information. In Step3, we finally identified a glioma core miRNA-gene module by integrating all the regulatory interaction between glioma survival related miRNAs and glioma core survival genes.

**Table 1:** Clinicopathological characteristics of patients in the training set, the testing set, and entire patient set.

Characteristic	Training set (N = 80)	Testing set (N = 80)	Entire patient set (N = 160)
Age (Mean±SD)	40.4±12.3	41.9±12.7	41,2±12.5
Gender			
Male (%)	48(60%)	48 (60%)	96 (60%)
Female (%)	32(40%)	32 (40%)	64 (40%)
Glioma histopathology (World Health Organization grading)			
Grade II (%)	32(40%)	31 (38.8%)	63 (39.4%)
Grade III (%)	16(20%)	17 (21.2%)	33 (20.6%)
Grade IV (%)	32(40%)	32 (40%)	64 (40%)
Patient survival			
Alive (%)	41(51.2%)	51 (63.7%)	92 (57.5%)
Deceased (%)	39(48.8%)	29 (36.3%)	68 (42.5%)
Survival days (Mean±SD)	652.5±328.3	707.4±333.2	679.9±332.8

#### Survival analysis

Two kinds of survival analysis were performed on miRNA (gene) signatures and module signatures. One is K-mean clustering method [39], the other is nearest centroid classification method [40]. For above two methods, glioma samples were both divided into two groups according to the expression value of the corresponding signature. The survival differences between two groups were assessed by Kaplan-Meier estimate, and compared using the log-rank test. We also performed Cox multivariate analysis to evaluate the contribution of other independent prognostic factors. The expression signature and other known factors were used in the multivariate analysis. In all survival analysis processes, a P-value<0.05 was considered to indicate a significant result.

#### The identification of survival pathways

For pathways from KEGG PATHWAY database, the more annotated glioma survival related genes (miRNA's target genes), the more association with the glioma survival. So hypergeometric distribution was utilized to evaluate the survival significance and the P-value was calculated for pathways as follows:
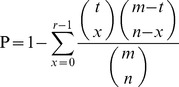



Where *m* was the number of the human whole genome, and *t* was the number of genes included in one pathway. The number of glioma survival related genes (or miRNA's target genes) was *n*, and *r* genes out of *n* genes were included in the pathway.

#### Pathway-based random walk

We developed pathway-based random walk (PbRW) method to identify glioma core survival genes based on the glioma survival related pathways. Firstly, we reconstructed the glioma survival related pathways graphically using R-based iSubpathwayMiner package [37] developed by our previous work. The reconstruction retained the raw information of these pathways, particularly for the pathway structure information. We then changed these pathway graphs into column-normalized adjacency matrices, which consisted of 0 and 1. For each adjacency matrix, we took the glioma survival related genes and miRNA's target genes as the seed nodes; and then utilized random walk algorithms to identify glioma core survival genes [41]. The formula of random walk algorithms is as follows:
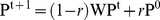



Where W is the column-normalized adjacency matrix of survival related pathway and 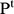 is a vector in which a node in the pathway matrix holds probability of finding itself in this process up to step t. In this study, the initial probability vector 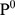 was constructed in such a way that equal probabilities were assigned to all seed nodes; the sum of their probabilities was equal to one. Additionally, the restart of the walker at each step is probability *r* (*r* = 0.7). Until the difference between 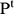 and 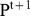 falls below 10^−6^, the probabilities will reach a steady state. Then, all genes in the pathway graph were ranked according to the values in the steady-state probability vector 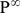.

## Results

### Identification of glioma core miRNA-gene survival module

#### Integration of miRNA and mRNA expression to identify glioma survival related pathways

For 80 patients of the training set, we performed Kaplan-Meier survival analysis on the sample-matched mRNA and miRNA expression data to identify glioma survival related genes and miRNAs. In this process, glioma samples were divided into two risk groups according to the mean expression value of the corresponding miRNA (gene) in each expression profiling, and P-values were calculated. A total of 115 miRNAs and 1962 genes were identified as glioma survival related miRNAs and genes, with P-values<0.001. For the 115 survival related miRNAs, we initially obtained their target genes which emerged from at least six of the eleven common miRNA target predicting datasets (see Materials and Methods). And for each survival related miRNA, we annotated its target genes into pathways from the KEGG database [36], and the pathway which was annotated by more target genes was more likely to be regulated by this miRNA. So hypergeometric distribution was utilized to identify significant biological pathways regulated by each survival related miRNAs with a strict cut-off of P-value<0.01. Similarly, we also identified 18 pathways which were significantly enriched by 1962 survival related genes. Among these pathways, 14 pathways were also regulated by more than one survival related miRNAs. When many pathways were considered, a high false positive discovery rate was likely to result, and we therefore calculated FDR corrected P-values for pathways in the identification procedure using the Benjamini-Hochberg FDR method (Table S1). The results showed that 14 common pathways also remained significant at the usual cut-off of FDR<0.15, suggesting a low false discovery rate. Finally, we regarded these 14 pathways as glioma survival related pathways, which were identified by integrating gene and miRNA expression level; most of these pathways were associated with occurrence of glioma tumor. Detailed information concerning the 14 glioma survival related pathways is given in Table S2.

#### Glioma survival related miRNAs and genes walking in the pathways to identify core survival module

Biological pathways provided topology structure information for miRNA-gene integrative analysis in glioma cell survival. So we developed a method named pathway-based random walk (PbRW) to identify more core survival genes at the pathway level. For each glioma survival related miRNA, we performed PbRW for all survival related pathways regulated by this miRNA. During this process, considering the joint impact of miRNA and gene level, we took glioma survival related genes and survival related miRNA's target genes as the seed nodes. Then a random walker started from these seed nodes to identify more core survival genes in the pathway structure. A detailed description of the PbRW method is shown in Materials and Methods. Following the PbRW method, all genes from each survival related pathway received a score; and a higher score indicated more survival association with glioma patients in this pathway. We utilized a stringent cutoff (top 3%) according to the score and thus obtained a set of glioma core survival genes from each pathway. Take an example, two membrane receptors (*ITGB* and *RTK*) were identified as glioma core survival genes from the focal adhesion pathway (Fig. S1). And for each survival related miRNAs, we combined all glioma core survival genes from all pathways it regulated, and constructed a miRNA-gene relationship. In this study, a total of 194 core survival genes were identified from all survival related pathways, and these genes were indirectly regulated by 34 survival related miRNAs. To systematically analyze glioma cell survival event, we further merged all the miRNA-gene relationship of 34 survival related miRNAs to construct a glioma core miRNA-gene module.

### Dissecting the glioma core survival module mediated by miRNAs

**Figure 2:**
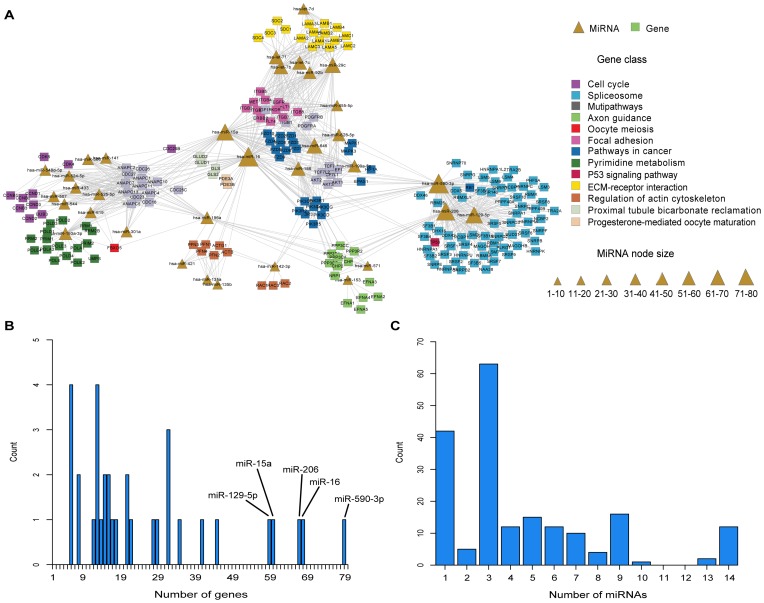
The glioma core survival module and distribution of miRNAs and genes within the module. (**A**). The triangles and rectangles in the core survival module correspond to miRNAs and genes, respectively. MiRNA node size is proportional to the degree of the node. Gene nodes are colored according to their categories, which include 13 pathway classes from KEGG pathway database. (**B**). Distribution of survival related miRNAs with respect to the number of their regulatory genes. (**C**). Distribution of core survival genes with respect to the number of times the gene is regulated by miRNAs.

The glioma core survival module consisted of 34 survival related miRNAs and 194 core survival genes (Figure 2). Of these survival related miRNAs, most regulated fewer core survival genes and five miRNAs regulated over 60 core survival genes. For example, miR-590-3p exhibited a regulatory relationship with 79 glioma core survival genes. MiR-16, miR-206, and miR-15a regulated 68, 67, and 60 core survival genes, respectively. Among these five hub miRNAs, miR-16 and miR-15a were found to be dysregulated in glioma [42]; moreover, they have performed cooperative regulatory functions in other cancers [43], [44]. Some genes in this survival module were similarly implicated in the occurrence and development of glioma, such as *ERBB2*, *ITGB3*, *EGFR*, and *MET* [45], [46], [47], [48]. As illustrated in Figure 2, all genes in the survival module were divided into 13 classes (12 pathways and 1 multi-pathway) according to the KEGG pathway classification. It was shown that some genes derived from multi-pathways, such as the *CDC* and *PDGFR* family genes, implying the pathway cross-talk in glioma survival process. The distribution of survival related miRNAs and their regulatory pathways are shown in Fig. S2. Similarly, miR-16 and miR-15a regulated over 7 survival related pathways. Some pathways, such as “Focal adhesion”, “Cell cycle” and “Pathways in cancer”, were also regulated by many miRNAs. And these pathways, especially focal adhesion, exhibited a close relationship with glioma tumor [1], [2], [49]. In a word, the core miRNA-gene survival module we identified was implicated in the gliomagenesis process at the miRNA, gene and pathway levels.

It was previously proposed that a higher-order structure is frequently observed in an integrated network. In our core miRNA-gene module, some miRNAs and genes were also closely connected and formed higher-order sub-modules. For mining the representative sub-modules as survival signatures for subsequent analysis, we further performed hierachical clustering on the bipartite miRNA-gene module. Based on the clustering result and intrinsic regulatory relationships, we identified a total of four sub-modules: moduleS1-moduleS4, in this study (Fig. S3). ModuleS3 was located in the center, whereas other three sub-modules were located in the peripheral part. In content, moduleS3 contained more hub miRNAs and genes as mentioned above. The miRNAs and genes in these four sub-modules are shown in Table 2.

**Table 2:** The detailed composition information of four sub-modules in the glioma core survival module.

Sub-module	MiRNA signature	Gene signature
ModuleS1	let-7b;let-7c;let-7f; miR-92b;let-7d;miR-29c	LAMA1-LAMA5;LAMB1-LAMB4; LAMC1-LAMC3;SDC1-SDC4
ModuleS2	miR-590-3p;miR-129-5p;miR-206	BUD31;DDX42;DDX46;DDX5;DHX15; HNRNPA1;HNRNPA1L2;HNRNPA3;HNRNPC; HNRNPK;HNRNPM;HNRNPU;LSM2-LSM7; MAGOH;MAGOHB;NAA38;NCBP1;NCBP2; PCBP1;PHF5A;PLRG1;RB1;PRPF40A;SNRPG;PRPF40B;RBM17;RBM25;RBM8A;RBMX; RBMXL1;SF3B14;SF3B1-SF3B5;SNRNP70; SNRPA1;SNRPB;SNRPB2;TRA2B;SNRPC; SNRPD1-SNRPD3;SNRPE;SNRPF; SR140;SRSF1-SRSF10;TRA2A
ModuleS3	miR-15a;miR-16; miR-646;miR-186; miR-455-5p;miR-628-5p	EGFR;ERBB2;FLT1;FLT4;FZD1-FZD10; IGF1R;ITGB1;ITGB3-ITGB8;KDR;MET; PDGFRA; PDGFRB
ModuleS4	miR-193a-3p;miR-141; miR-544;miR-507; miR-524-5p;miR-586; miR-433;miR-619; miR-548d-5p; miR-525-5p;miR-301a	ANAPC1;ANAPC10;ANAPC11;ANAPC13; ANAPC2;ANAPC4;ANAPC5;ANAPC7;BUB3; CCNB1-CCNB3;CCND1-CCND3;CDC16; CDC23;CDC25B;CDC25C;CDC26;CDC27; CDK4;CDK6;FBXO5;POLA1;POLA2;POLE; POLD1-POLD4;POLE2-POLE4;PRIM1; PRIM2;RRM1;RRM2;RRM2B;UMPS

### Prediction power of the glioma core miRNA-gene survival module

#### Validation of the sub-modules for survival prediction by the testing set

**Figure 3:**
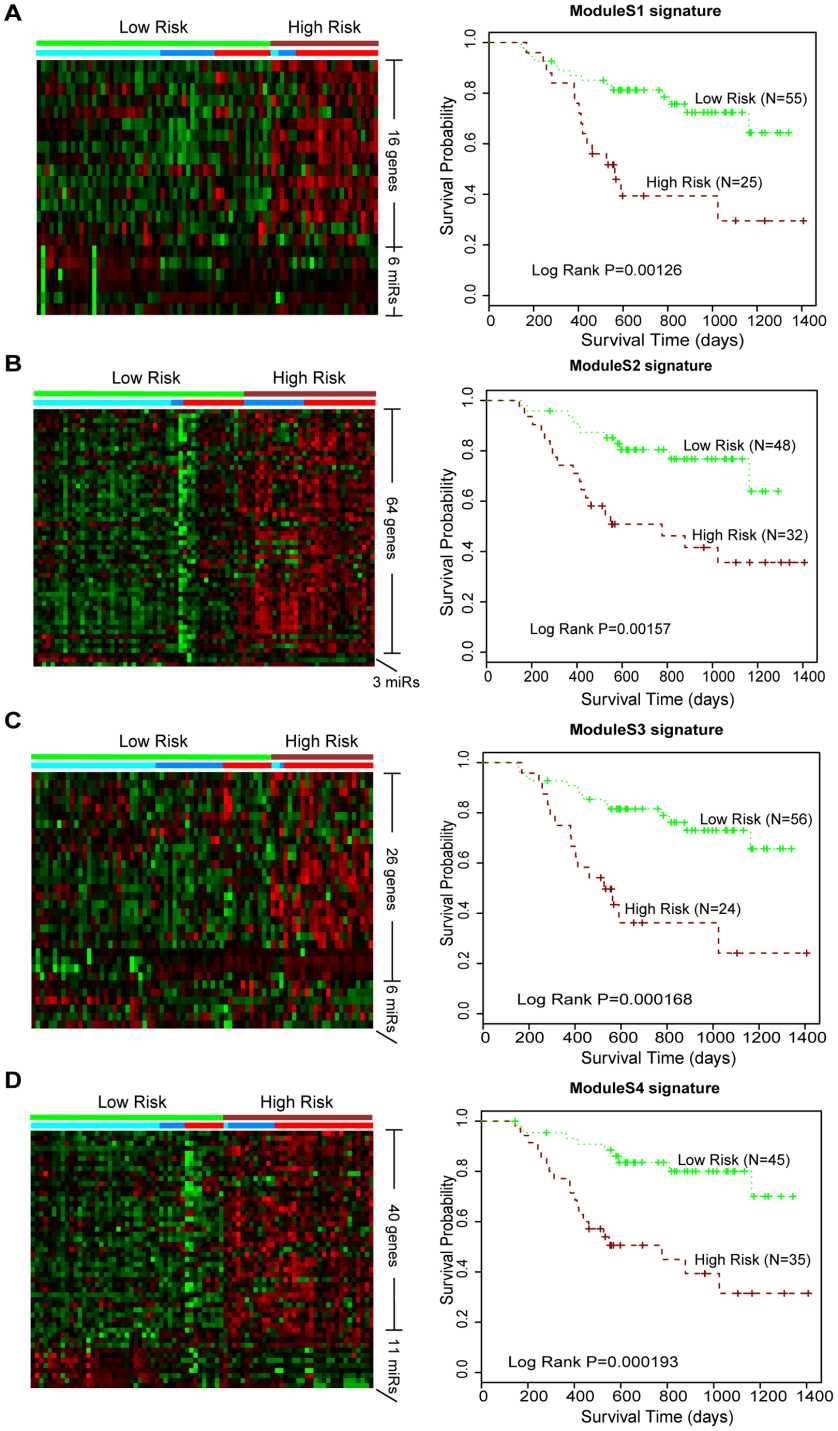
Module signatures predict glioma patient clinical outcome. (**A-D**). Four sub-modules (moduleS1-S4). K-mean clustering representation of module signatures in the 80 glioma patients of the testing set. The columns represent tumor samples and rows represent genes and miRNAs in corresponding module. Red indicates high relative expression levels, whereas green low levels. Horizontal bars above the heat map indicate the grade status and class of the patient (cyan, deepblue and red box indicated grade II, III and IV; green and darkred box indicated low-risk and high-risk class). The low-risk and high-risk groups were derived from K-mean clustering (K = 2) and estimated by Kaplan-Meier survival analysis. P-values were calculated by the log-rank test.

Four sub-modules identified above with proper size were representative of the entire module as survival signatures, and their prediction power were further evaluated using the testing set. We firstly merged sample-matched miRNA and mRNA expression profiles after row and column normalization and then performed K-mean clustering (K = 2) to achieve merged expression profiling with four sub-module signatures. Finally, Kaplan-Meier survival analysis was applied to evaluate the corresponding signature's prediction effect. As shown in Figure 3, these four sub-modules were all significantly associated with the survival status of 80 glioma patients in the testing set, with P-value = 0.0013, 0.0016, 0.0002, and 0.0002, respectively. Moreover, using the raw miRNA (gene) expression profiles, the corresponding miRNA and gene signatures of four sub-modules were also evaluated. Interestingly, all these miRNA and gene signatures correlated with glioma patient survival with P-values≤0.001, strengthening the clinical prediction value of our sub-module signatures (Table 3). Furthermore, the prediction power of these four sub-modules in patient subgroups (high-grade glioma patients, n = 49) from the testing set were evaluated. In all four sub-modules, moduleS3 signature also exhibited strong prediction power for high-grade glioma patients (module signature, P-value = 0.0062; gene signature, P-value = 0.0163).

**Table 3:** The P-value performance of four sub-modules using Kaplan-Meier survival analysis in the testing set.

	Testing set (Grade II,III and IV; n = 80)				Testing set (Grade III and IV; n = 49)			
	ModuleS1	ModuleS2	ModuleS3	ModuleS4	ModuleS1	ModuleS2	ModuleS3	ModuleS4
**Module signature**	0.0013	0.0016	0.0002	0.0002	0.2440	0.6710	0.0062	0.1600
**MiRNA signature**	0.0068	0.0003	0.0001	0.0000	0.6790	0.5070	0.1710	0.0010
**Gene signature**	0.0000	0.0009	0.0010	0.0000	0.0929	0.5180	0.0163	0.1170

ModuleS3 signature from the core miRNA-gene module displayed the best performance in the survival prediction. To test the prediction robustness of module signature, we further performed another survival analysis method based on nearest centroid classification [40]. Using the expression signature, we performed K-mean clustering on the testing set except one sample to form two groups, one high-risk group and one low-risk group. Then the external sample was assigned to high-risk or low-risk group according to the nearest centroid classification rule. After all samples were assigned to risk groups, K-M estimate was finally used to evaluate the signature's prediction power. As shown in Fig. S4, the moduleS3 signature also predicted clinical outcome of patients in the testing set (P-value = 0.0049) and the high-grade subgroup (P-value = 0.0056). From the moduleS3 signature, only a few miRNAs and genes were associated with glioma patient survival, suggesting the signature set owned prediction power not individual component (Table S3).

Moreover, to test whether our expression signature predicted patient survival independently of other prognostic factors in our cohort, we also performed multivariate analysis (Table S4). It was shown that all expression signatures predicted outcome independently of other factors such as age, gender and IDH1 mutation. Notably, the survival prediction of 26-gene signature was also independent of patient stage, a known prognostic factor, with P-value = 0.034. Taken together, the core miRNA-gene module was closely related with glioma survival. Especially, one core sub-module (moduleS3) had strong prediction power for clinical outcome of glioma patients.

#### Revalidation of moduleS3 signature for survival prediction by glioma independent datasets

**Figure 4:**
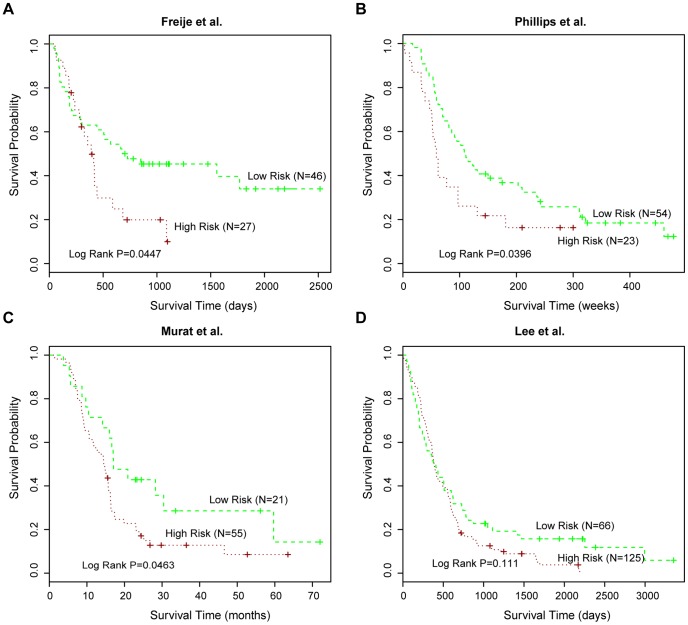
The gene signature from moduleS3 predicts clinical outcome of glioma datasets from GEO. The glioma datasets were respectively extracted from the studies of (**A**) Freije et al., (**B**) Phillips et al., (**C**) Murat et al., and (**D**) Lee et al.. The significance of clinical outcome difference between the low-risk and high-risk groups was estimated by K-M survival analysis. P-values were calculated by the log-rank test.

To further validate the prediction power of moduleS3 signature, we collected all public glioma expression datasets with available survival information from the GEO database [23]. According to Dobbin and Simon [50], the number of samples required for testing prognostic signatures was proximately 50 or above for a general expression dataset. Thus, we chose n = 50 as our minimum sample size requirement and four gene expression datasets with corresponding survival information from studies by Freije et al. [16], Phillips et al. [20], Murat et al. [21], and Lee et al. [22] were obtained. Gene expression signature of moduleS3 was applied to predict clinical outcome of samples within these datasets. For two WHO grade III and IV datasets (Freije et al. and Phillips et al.), 26-gene expression signature exhibited significant prediction power for glioma patients, with P-values<0.05 (Figure 4A, B). For other two grade IV datasets (Murat et al. and Lee et al.), the expression signature was also associated with GBM patient survival, with P-values = 0.0463 and 0.111, respectively (Figure 4C, D). Next, we also obtained sample-matched miRNA and mRNA expression data of GBM from TCGA database. The TCGA provided public glioma multi-dimensional expression data similar to our dataset. Then survival analysis based on K-mean clustering were performed on miRNAs, genes and moduleS3 signature. As shown in Fig. S5, the 26 genes in moduleS3 exhibited a marginal significant association with GBM survival (P-value = 0.0663), and could predict the clinical outcome of patients who had the survival time longer than two years (P-value = 0.019), further strengthening its survival prediction power.

**Figure 5:**
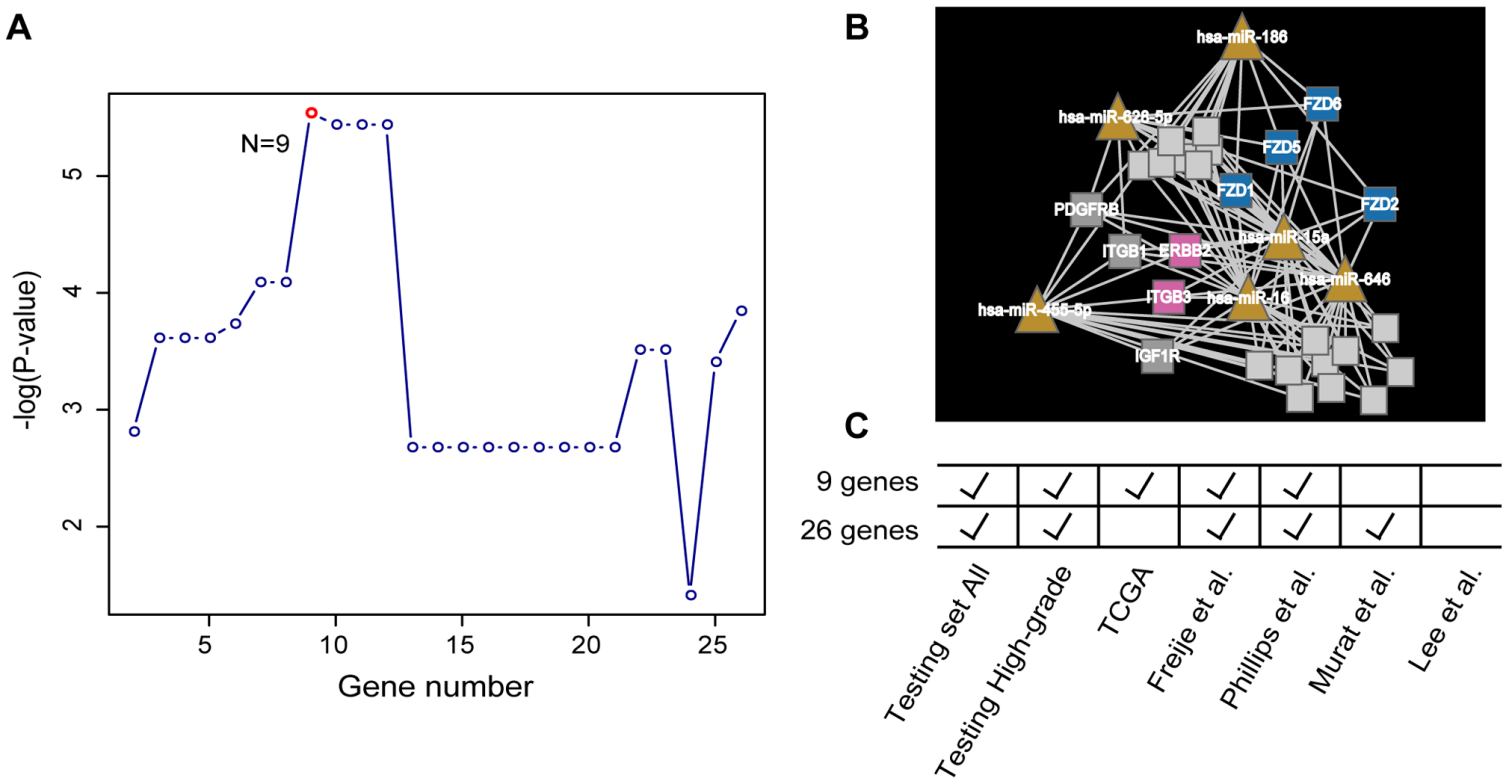
The survival prediction performance of the 9-gene signature from moduleS3. (**A**). The identification of top 9 genes from the moduleS3. (**B**). The 9 genes in moduleS3. Gene nodes were colored according to the gene class colors used in Figure 2. (**C**). The survival prediction power comparison between 9-gene and 26-gene signatures.

The 26-gene signature of moduleS3 exhibited robust power to predict glioma patient clinical outcome in many datasets mentioned above. We further analyzed the gene signature to determine whether a subset could also be used to predict patient survival. Among the 26-gene signature, 3 genes (*KDR*, *PDGFRA*, and *IGF1R*) were target genes of survival related miRNAs, and 4 genes (*PDGFRB*, *FZD6*, *ITGB1* and *IGF1R*) were most significantly associated with patient survival (P-values<0.001). However, unlike the 26-gene signature, the corresponding target genes and significant genes in moduleS3 did not consistently correlate with glioma patient survival in most cases (data not shown). In addition, we developed an optimization method for the 26 genes; we first ranked these genes according to their survival significance, and regarded the top n genes (n from 2 to 25) as subset signatures. As shown in Figure 5, the top 9 genes exhibited the best survival performance. After other survival verification, the 9-gene signature also predicted the clinical outcome of glioma patients in the testing set, high-grade subgroup, and three independent datasets.

## Discussion

We systematically analyzed glioma survival by considering joint impact of miRNA and gene at the pathway level. During the analytical process, we put glioma survival related miRNAs and genes walking in the pathways to identify glioma core survival genes, and then constructed a glioma miRNA-gene module. Following survival verification using the testing set and independent datasets, one core sub-module, especially gene signature included, was shown to be a potential predictor for glioma patient clinical outcome.

In this study, we divided all the glioma samples randomly into a training set and a testing set without significant difference in clinicopathologic features. The single training-testing partition may not provide the most robust signature results. So we first permutated partial samples (n = 5, 10, 15 and 20) based on original training and testing sets and performed our integrated analytical method to identify glioma miRNA-gene module results. And a recurrence ratio was defined to show the ratio of miRNAs (genes) of our original module that were also identified in the new results. As shown in Fig. S6, there were strong robust results for glioma miRNA-gene module and core sub-module3, especially the 26 genes in moduleS3 even when n = 20. Also, the gene signatures exhibited strong predictive power in clinical outcome of glioma patients from four additional datasets (Figure 5). To further test the robustness of these expression signatures, we performed another random permutation analysis named shuffle-and-split analysis; we shuffled all the 160 glioma samples and randomly splitted into two pairs of training and testing sets. We repeated this process a total of 500 times. The results showed that the 26-gene and 9-gene signatures also had high recurrence ratio, further verifying the robustness of our expression signatures (Fig. S7).

By integrating sample-matched miRNA and gene expression, we identified 14 glioma survival related pathways using the hypergeometric distribution method, which had more advantage for strong significant pathway identification. Among these survival related pathways, “Focal adhesion”, “Cell cycle”, “Pyrimidine metabolism”, “Pathways in cancer”, “ECM-receptor interaction”, and “P53 signaling pathway” were all known to be related to the occurrence and metastasis of glioma tumor (Table S2). Notably, cell-matrix adhesion played an essential role in important biological processes, including cell survival, proliferation and motility [2]. It has been previously reported that the focal adhesion is associated with glioma tumors, and that adhesion receptors promote glioma cell migration and invasion [2], [49]. In our core survival module, “Focal adhesion” pathway was involved in the moduleS3, supporting its sound survival predictive effect. Moreover, some disease pathways, such as “Colorectal cancer” and “Small cell lung cancer”, were also identified; thus, there might exist some common biological mechanism or disease genes shared among these diseases and glioma. In a word, these pathways were of biological importance and PbRW was further developed on them for mining core survival factors.

During the PbRW method to identify glioma core survival genes, we simultaneously considered topology information derived from pathway structure and the joint impact of two levels, miRNA and mRNA expression. We regarded the glioma survival related genes and survival related miRNA's targets as seed genes, and equal probabilities were assigned to all seeds in this process. Then a random walker was walking step by step in pathway structure to identify core survival genes, which were drove by survival related miRNAs and genes. The PbRW method accounted for both number and length of multiple paths connecting two nodes in the pathway structure. Furthermore, this method also allowed the algorithm to restart walk at the seed nodes in every iteration, which enabled the choice of a trade-off between the exploitation of local and global pathway structure. The benefits of random walk algorithm for node scoring have been discussed and are currently utilized for disease-gene prioritization [41].

From the glioma core miRNA-gene module, four representative sub-modules were identified and evaluated using the testing set and independent datasets. All four module signatures were significantly associated with clinical outcome of patients with grade II to IV. Notably, when applied to high-grade subgroup (grade III and IV), moduleS3 also had a prognostic value for glioma patients. This implied the assumption that a “low grade” glioma like behaviour existed in high-grade tumor which would be associated with better outcome. And in the multivariate analysis with patient stage, the 26-gene signature was an independent predictor of patient survival with P-value = 0.034. As mentioned above, many pathways including focal adhesion were involved in moduleS3. So the integration of multi-pathways might have an implication in discriminating the clinical outcome of high-grade glioma patients.

By independent survival verification, our 26-gene expression signature could predict the clinical outcome for glioma patients from GEO and TCGA databases. Furthermore, we also performed another survival analysis method [40] to test the expression signature's prediction robustness. In these results, the 26-gene expression signature was significantly associated with the patient survival in the TCGA data set with P-value = 0.018 (Fig. S8 A). For three of four GEO data sets, our signature also displayed the prediction power for patient clinical outcome (P-values<0.05), showing our expression signature's robustness in survival prediction. In this study, we looked at survival across glioma grades to identify robust expression signatures, which displayed prediction power in high-grade and GBM cohorts. Also, the grade-specific differences in gene/miRNA expression and survival difference between different grades were still of importance, and will be considered in future study.

It is important to predict therapy responsiveness and to spare certain patients from unnecessary adjuvant therapies that have adverse side effects. For example, GBM patients with MGMT promoter methylation who were treated with temozolomide had a median survival of 21.7 months. In contrast, patients without MGMT promoter methylation had a significantly shorter median survival of only 12.7 months [51]. Thus, we further investigated the relationship between our expression signatures and chemotherapy treatment. Of all the datasets analyzed above, only two datasets (TCGA and study of Murat et al.) contained patient treatment information and TCGA dataset with adequate samples (>50) could be utilized for further analysis. Temozolomide is the most common chemotherapy drug for glioma clinical treatment. So we extracted temozolomide-treated samples from TCGA dataset and examined the predictive capacity of 26-gene and 9-gene signatures for these samples; two sample groups were formed: high-risk and low-risk groups. As shown in Fig. S9, there was a significantly different outcome between the two predicted groups, showing that our expression signatures were also prognostic in temozolomide treated patients (n = 194; P-values<0.05). Moreover, the multivariable analysis showed that these signatures predicted patient survival outcome independently of gender and KPS score (Table S4).

We integrated high-throughput miRNA, mRNA expression, and pathway structure to systematically identify a glioma survival module, among which 26 gene signature was capable of predicting patient clinical outcome. Sample-matched miRNA and mRNA expression data with patient survival information has recently been developed; thus, further validation of the expression signature will help strengthen its clinical value. In the current study, our findings are potentially useful for understanding the gliomagenesis and identifying expression signatures for clinical outcome prediction.

## Supporting Information

Figure S1 **Focal adhesion (path: 04510): a pathway example.** Node colors: blue node, glioma survival related genes but not survival related miRNA targets; green node, glioma survival related miRNA targets but not survival related genes; yellow node, both glioma survival related genes and miRNA targets. In this pathway, membrane receptor *ITGB* received the highest score (0.04925) overall. Another receptor gene *KDR* received the second high score (0.04473).
(PDF)

Figure S2 **Distribution of glioma survival related miRNAs and their regulatory survival pathways.** (**A**). Distribution of survival related miRNAs with respect to number of their regulatory pathways. (**B**). Distribution of glioma survival related pathways with respect to the number of times the pathway is regulated by miRNAs.
(PDF)

Figure S3 **Hierarchical clustering on the glioma core survival module and four representative sub-modules.** (**A**). Hierarchical clustering on the glioma core survival module using the correlation (uncentered) and complete linkage method in the Cluster3 software package and JavaTreeView imaging software. The corresponding cell was colored red if there was an edge between the miRNA and gene. Gene labels were colored according to the gene class colors used in Figure 2. The bars above the gene and miRNA labels showed four sub-modules (yellow, cyan, pink and grey indicated moduleS1 to S4). (**B**). Four representative sub-modules (moduleS1-S4) in the glioma core survival module.
(PDF)

Figure S4 **The moduleS3 signature predicts the clinical outcome of samples from the Testing set using nearest centroid classification method.** (**A**). Testing set grade II/III/IV (**B**). Testing set high-grade sub-group.
(PDF)

Figure S5 **The 26-gene signature from moduleS3 predicts the clinical outcome of samples from TCGA dataset.**
(PDF)

Figure S6 **The recurrence ratio of glioma miRNA-gene module results after partial sample perturbation analysis.**
(PDF)

Figure S7 **The shuffle-and-split analysis of our expression signatures.** The recurrence ratio of core gene signatures in 500 random shuffle-and-split analysis. The genes which were colored red belonged to the 9-gene signature (see Figure 5).
(PDF)

Figure S8 **The 26-gene signature predicts the clinical outcome of glioma samples using nearest centroid classification method.** (**A**). TCGA dataset and the study of (**B**) Freije et al., (**C**) Phillips et al., (**D**) Lee et al., and (**E**) Murat et al.
(PDF)

Figure S9 **The 26-gene signature from moduleS3 predicts the clinical outcome of temozolomide-treated samples from TCGA.**
(PDF)

Table S1 The biological pathways enriched by glioma survival related genes (survival related miRNA's target genes).
(XLS)

Table S2 The detailed information of glioma survival related pathways.
(DOC)

Table S3 The P-value performance of each miRNA (gene) from moduleS3 in the testing set.
(XLS)

Table S4 Univariable and multivariable cox regression analysis of our expression signatures.
(XLS)
