# Supplementary figures and images for: Identification of miRNA-Mediated Core Gene Module for Glioma Patient Prediction by Integrating High-Throughput miRNA, mRNA Expression and Pathway Structure
Source: PLoS One. 2014 May 8;9(5):e96908. doi: 10.1371/journal.pone.0096908 (PMC4014552; doi:10.1371/journal.pone.0096908)

# FOCAL ADHESION

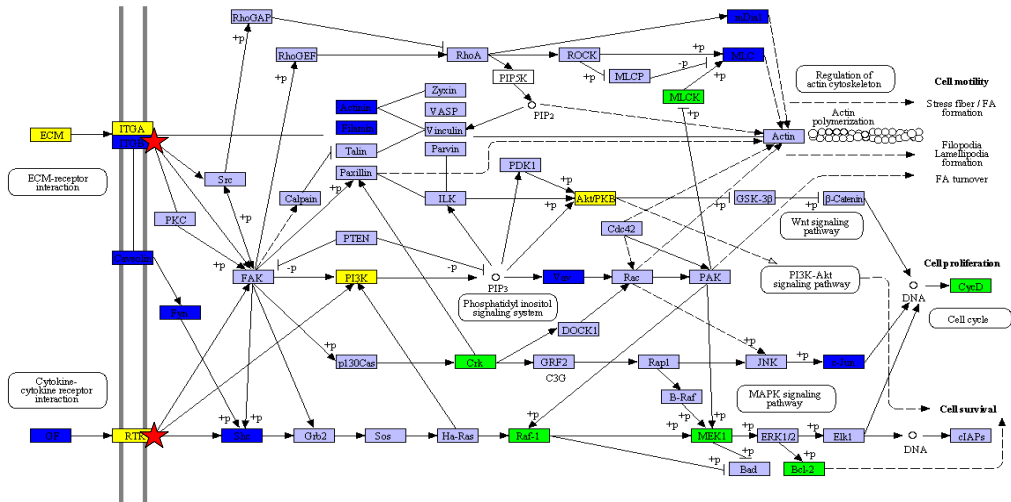

Supplement: Figure S1 — Focal adhesion (path: 04510): a pathway example. Node colors: blue node, glioma survival related genes but not survival related miRNA targets; green node, glioma survival related miRNA targets but not survival related genes; yellow node, both glioma survival related genes and miRNA targets. In this pathway, membrane receptor ITGB received the highest score (0.04925) overall. Another receptor gene KDR received the second high score (0.04473). (PDF) [file pone.0096908.s001.pdf]

**A**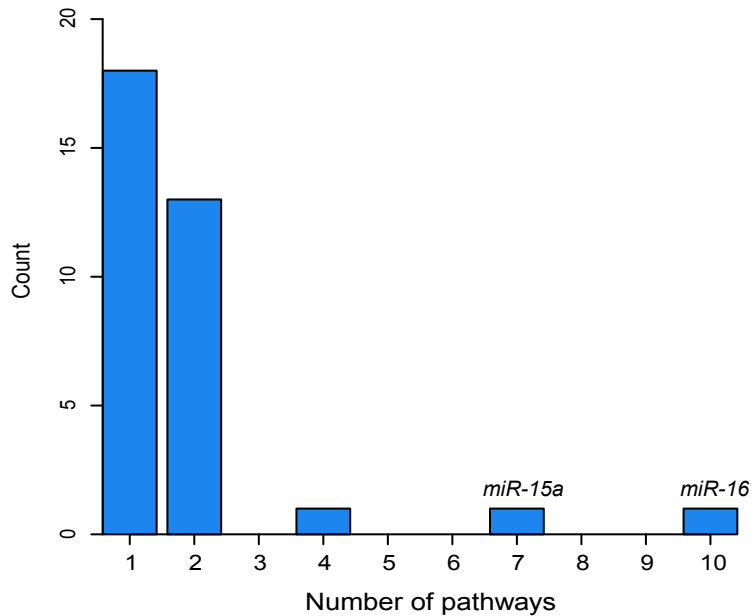**B**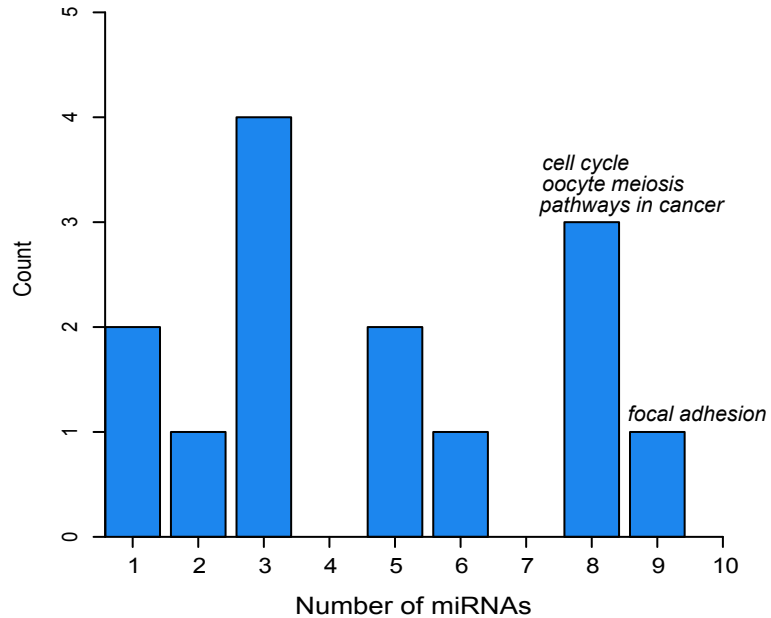

Supplement: Figure S2 — Distribution of glioma survival related miRNAs and their regulatory survival pathways. (A). Distribution of survival related miRNAs with respect to number of their regulatory pathways. (B). Distribution of glioma survival related pathways with respect to the number of times the pathway is regulated by miRNAs. (PDF) [file pone.0096908.s002.pdf]

# A

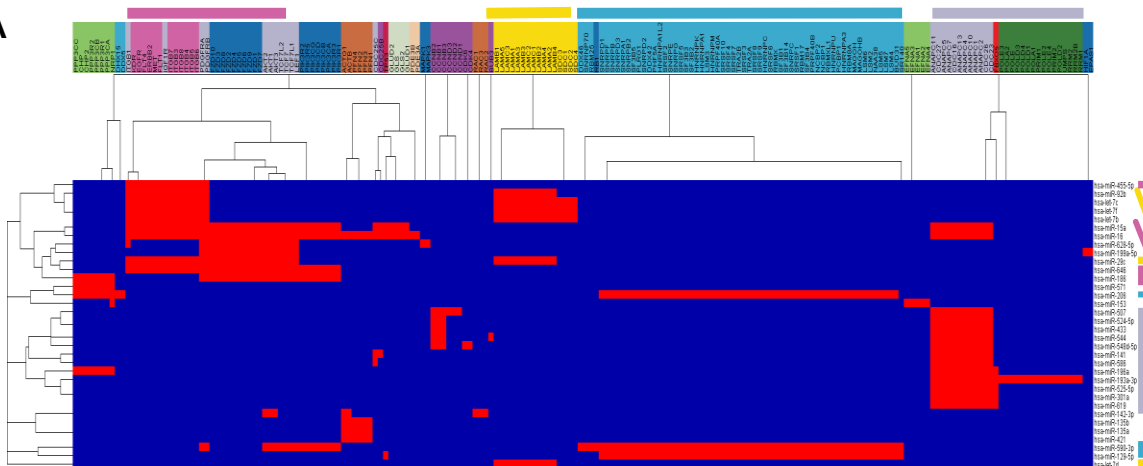

# B

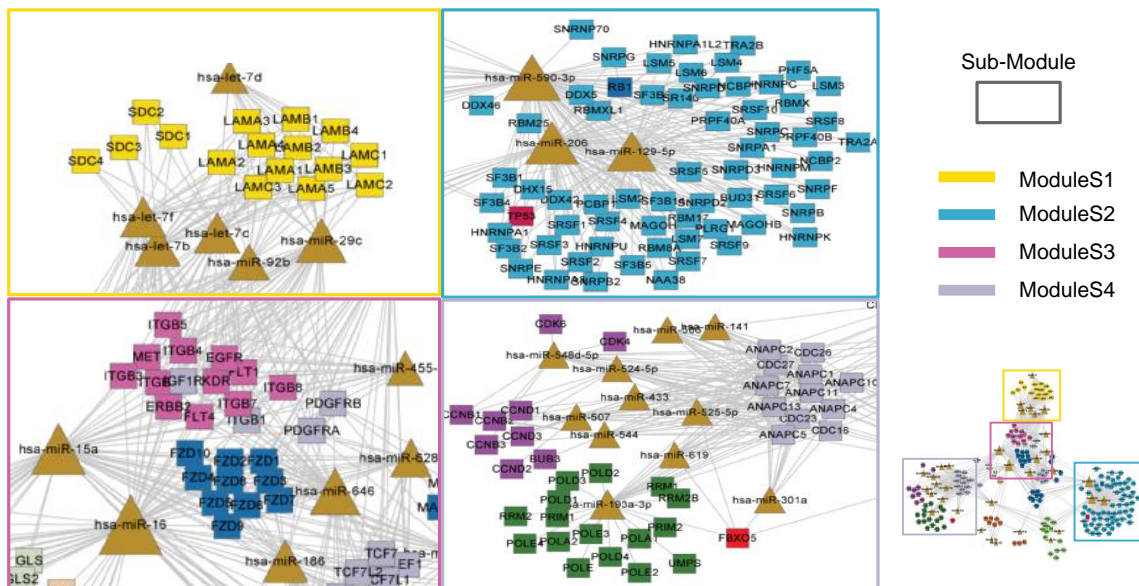

Supplement: Figure S3 — Hierarchical clustering on the glioma core survival module and four representative sub-modules. (A). Hierarchical clustering on the glioma core survival module using the correlation (uncentered) and complete linkage method in the Cluster3 software package and JavaTreeView imaging software. The corresponding cell was colored red if there was an edge between the miRNA and gene. Gene labels were colored according to the gene class colors used in Figure 2. The bars above the gene and miRNA labels showed four sub-modules (yellow, cyan, pink and grey indicated moduleS1 to S4). (B). Four representative sub-modules (moduleS1-S4) in the glioma core survival module. (PDF) [file pone.0096908.s003.pdf]

## 26-gene Signature in TCGA

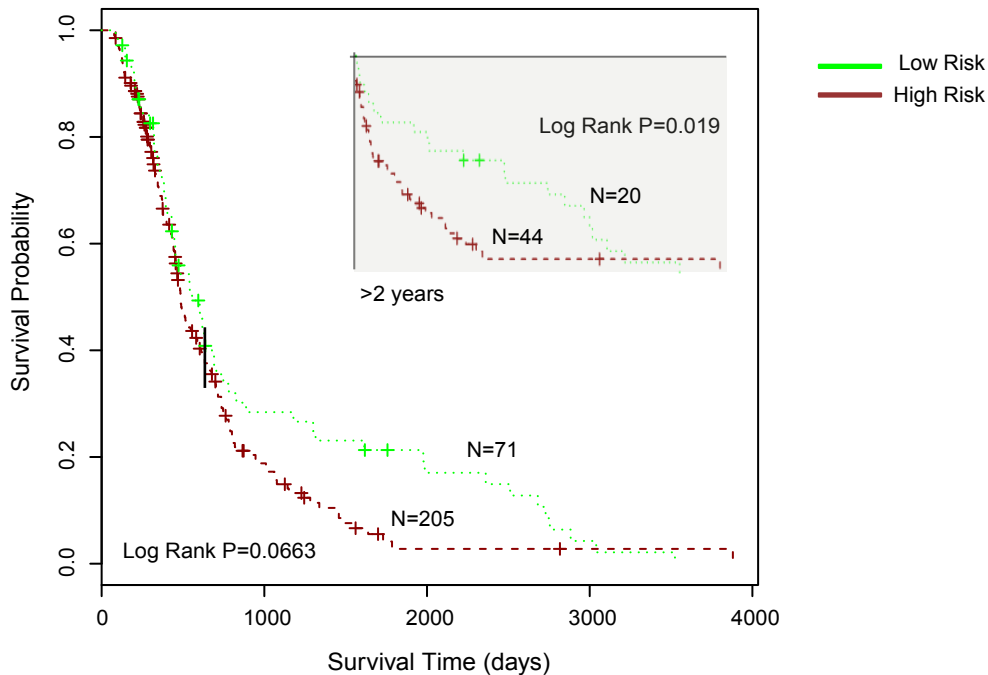

Supplement: Figure S5 — The 26-gene signature from moduleS3 predicts the clinical outcome of samples from TCGA dataset. (PDF) [file pone.0096908.s005.pdf]

## Sample perturbation result

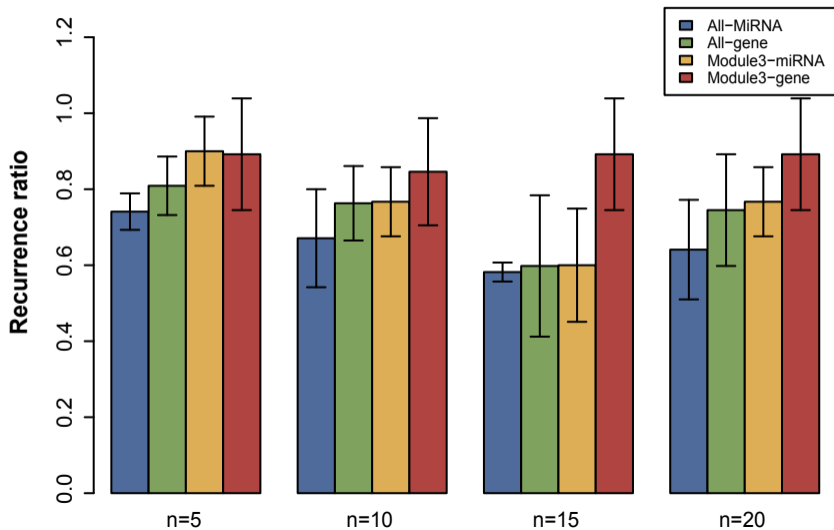

Supplement: Figure S6 — The recurrence ratio of glioma miRNA-gene module results after partial sample perturbation analysis. (PDF) [file pone.0096908.s006.pdf]

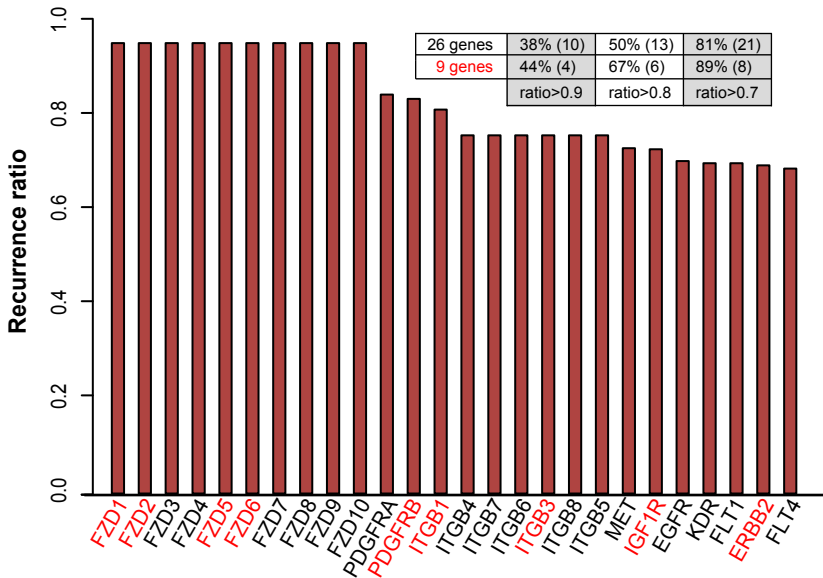

Supplement: Figure S7 — The shuffle-and-split analysis of our expression signatures. The recurrence ratio of core gene signatures in 500 random shuffle-and-split analysis. The genes which were colored red belonged to the 9-gene signature (see Figure 5). (PDF) [file pone.0096908.s007.pdf]

**26-gene Signature in TCGA temozolomide-treated**

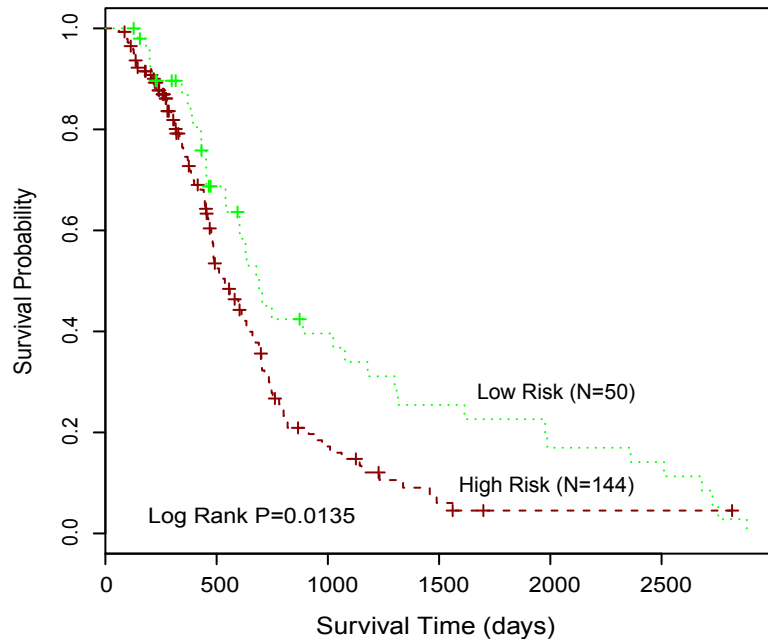

**9-gene Signature in TCGA temozolomide-treated**

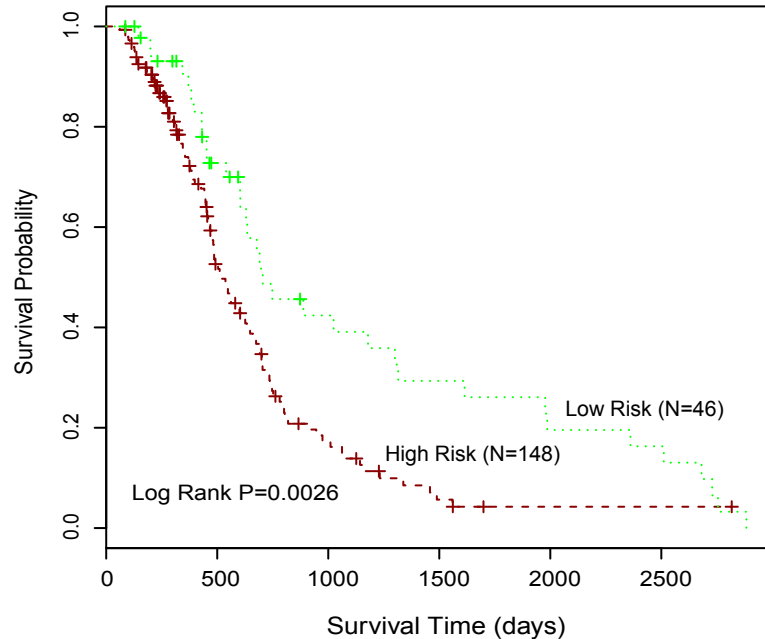

Supplement: Figure S9 — The 26-gene signature from moduleS3 predicts the clinical outcome of temozolomide-treated samples from TCGA. (PDF) [file pone.0096908.s009.pdf]
