# Supplementary material for: Identification of miRNA-Mediated Core Gene Module for Glioma Patient Prediction by Integrating High-Throughput miRNA, mRNA Expression and Pathway Structure
Source: PLoS One. 2014 May 8;9(5):e96908. doi: 10.1371/journal.pone.0096908 (PMC4014552; doi:10.1371/journal.pone.0096908)
Supplement: Figure S4 — The moduleS3 signature predicts the clinical outcome of samples from the Testing set using nearest centroid classification method. (A). Testing set grade II/III/IV (B). Testing set high-grade sub-group. (PDF) [file pone.0096908.s004.pdf]

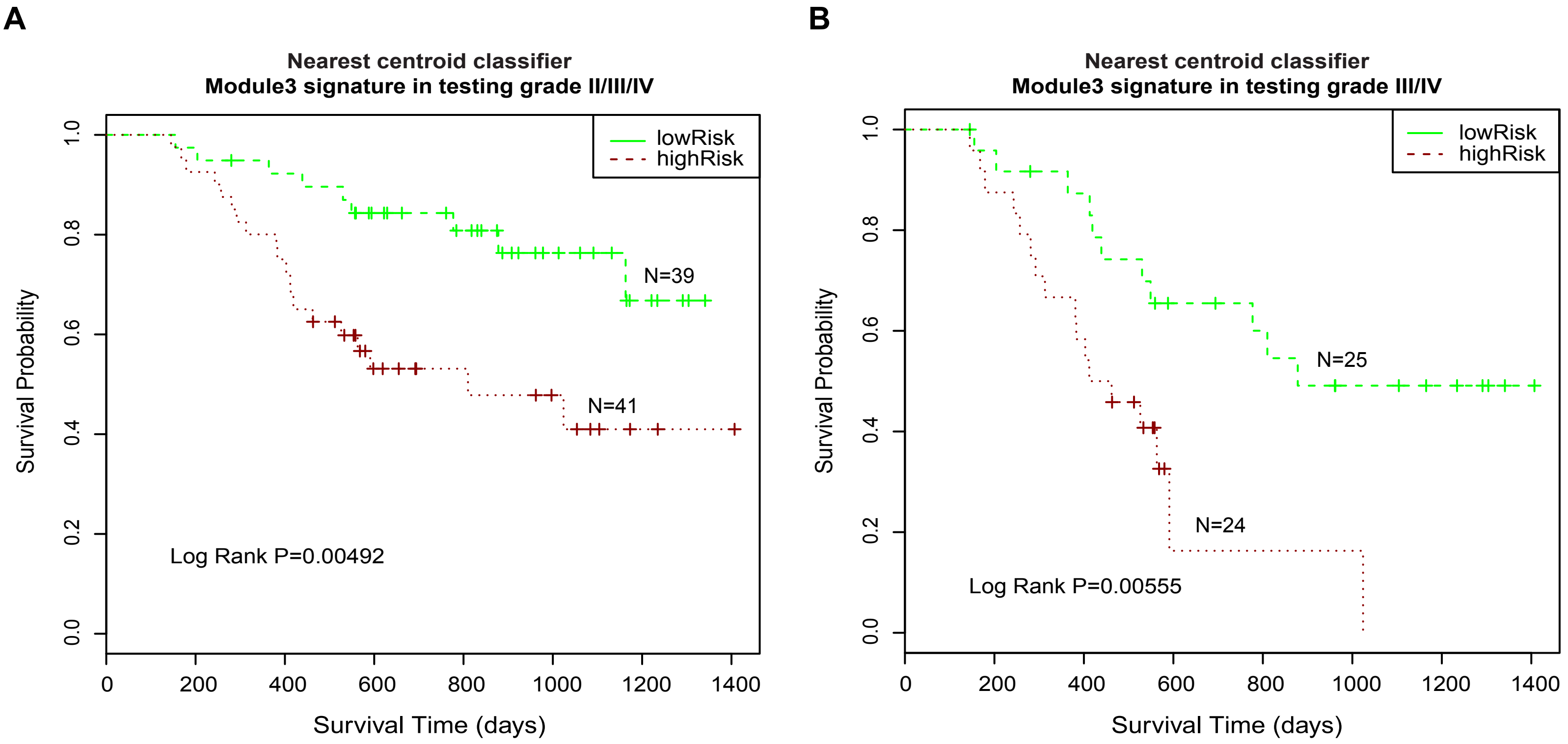
A
Nearest centroid classifier
Module3 signature in testing grade II/III/IV
lowRisk
highRisk
N=39
N=41
Log Rank P=0.00492
Survival Probability
Survival Time (days)
0.0
0.2
0.4
0.6
0.8
1.0
0
200
400
600
800
1000
1200
1400
B
Nearest centroid classifier
Module3 signature in testing grade III/IV
lowRisk
highRisk
N=25
N=24
Log Rank P=0.00555
Survival Probability
Survival Time (days)
0.0
0.2
0.4
0.6
0.8
1.0
0
200
400
600
800
1000
1200
1400
