# Supplementary material for: Identification of miRNA-Mediated Core Gene Module for Glioma Patient Prediction by Integrating High-Throughput miRNA, mRNA Expression and Pathway Structure
Source: PLoS One. 2014 May 8;9(5):e96908. doi: 10.1371/journal.pone.0096908 (PMC4014552; doi:10.1371/journal.pone.0096908)
Supplement: Figure S8 — The 26-gene signature predicts the clinical outcome of glioma samples using nearest centroid classification method. (A). TCGA dataset and the study of (B) Freije et al., (C) Phillips et al., (D) Lee et al., and (E) Murat et al. (PDF) [file pone.0096908.s008.pdf]

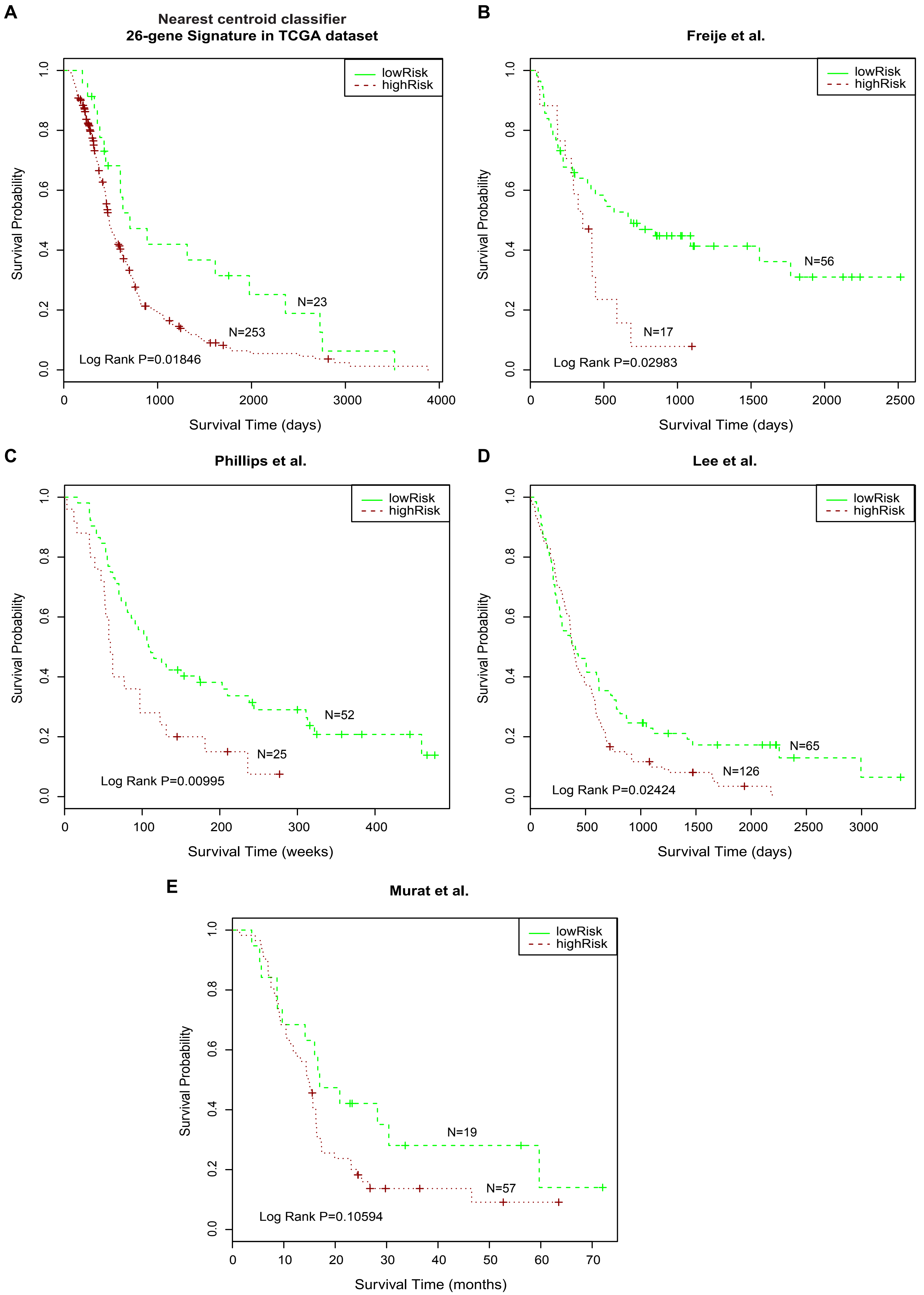
A
Nearest centroid classifier
26-gene Signature in TCGA dataset
lowRisk
highRisk
N=23
N=253
Log Rank P=0.01846
Survival Probability
Survival Time (days)
B
Freije et al.
lowRisk
highRisk
N=56
N=17
Log Rank P=0.02983
Survival Probability
Survival Time (days)
C
Phillips et al.
lowRisk
highRisk
N=52
N=25
Log Rank P=0.00995
Survival Probability
Survival Time (weeks)
D
Lee et al.
lowRisk
highRisk
N=65
N=126
Log Rank P=0.02424
Survival Probability
Survival Time (days)
E
Murat et al.
lowRisk
highRisk
N=19
N=57
Log Rank P=0.10594
Survival Probability
Survival Time (months)
