# Supplementary material for: Identification of miRNA-Mediated Core Gene Module for Glioma Patient Prediction by Integrating High-Throughput miRNA, mRNA Expression and Pathway Structure
Source: PLoS One. 2014 May 8;9(5):e96908. doi: 10.1371/journal.pone.0096908 (PMC4014552; doi:10.1371/journal.pone.0096908)
Supplement: Table S2 — The detailed information of glioma survival related pathways. (DOC) [file pone.0096908.s011.doc]

**The detailed information of glioma survival related pathways.**

| **PathwayId** | **PathwayName** | **miRNANumber** | **miRNAName** | **Reference** |
| --- | --- | --- | --- | --- |
| path:04510 | Focal adhesion | 9 | miR-646; miR-15a; miR-92b; miR-29c; let-7b; miR-16; let-7c; let-7f; miR-455-5p |  |
| path:04110 | Cell cycle | 8 | miR-507; miR-544; miR-524-5p; miR-15a; miR-141; miR-16; miR-433; miR-548d-5p |  |
| path:05200 | Pathways in cancer | 8 | miR-15a; miR-16; miR-628-5p; miR-29c; miR-590-3p; miR-646; miR-199a-5p; miR-186 | Cancer |
| path:04114 | Oocyte meiosis | 8 | miR-196a; miR-193a-3p; miR-525-5p; miR-15a; miR-301a; miR-619; miR-16; miR-586 | —— |
| path:04512 | ECM-receptor interaction | 6 | miR-29c; let-7d; let-7b; miR-92b; let-7c; let-7f |  |
| path:04360 | Axon guidance | 5 | miR-196a; miR-153; miR-571; miR-206; miR-186 |  |
| path:04810 | Regulation of actin cytoskeleton | 5 | miR-16; miR-421; miR-135b; miR-135a; miR-142-3p |  |
| path:05222 | Small cell lung cancer | 3 | miR-29c; miR-16; miR-628-5p | Cancer |
| path:05210 | Colorectal cancer | 3 | miR-15a; miR-16; miR-142-3p | Cancer |
| path:03040 | Spliceosome | 3 | miR-206; miR-129-5p; miR-590-3p |  |
| path:04115 | p53 signaling pathway | 3 | miR-15a; miR-16; miR-129-5p |  |
| path:04964 | Proximal tubule bicarbonate reclamation | 2 | miR-15a; miR-16 | —— |
| path:00240 | Pyrimidine metabolism | 1 | miR-193a-3p |  |
| path:04914 | Progesterone-mediated oocyte maturation | 1 | miR-16 | —— |

Reference

1. Lee J, Borboa AK, Chun HB, Baird A, Eliceiri BP (2010) Conditional deletion of the focal adhesion kinase FAK alters remodeling of the blood-brain barrier in glioma. Cancer Res 70: 10131-10140.

2. Golubovskaya VM, Huang G, Ho B, Yemma M, Morrison CD, et al. (2013) Pharmacologic blockade of FAK autophosphorylation decreases human glioblastoma tumor growth and synergizes with temozolomide. Mol Cancer Ther 12: 162-172.

3. Srikanth M, Das S, Berns EJ, Kim J, Stupp SI, et al. (2013) Nanofiber-mediated inhibition of focal adhesion kinase sensitizes glioma stemlike cells to epidermal growth factor receptor inhibition. Neuro Oncol 15: 319-329.

4. Pennarun G, Granotier C, Gauthier LR, Gomez D, Hoffschir F, et al. (2005) Apoptosis related to telomere instability and cell cycle alterations in human glioma cells treated by new highly selective G-quadruplex ligands. Oncogene 24: 2917-2928.

5. Maddika S, Ande SR, Panigrahi S, Paranjothy T, Weglarczyk K, et al. (2007) Cell survival, cell death and cell cycle pathways are interconnected: implications for cancer therapy. Drug Resist Updat 10: 13-29.

6. Vadlamuri SV, Media J, Sankey SS, Nakeff A, Divine G, et al. (2003) SPARC affects glioma cell growth differently when grown on brain ECM proteins in vitro under standard versus reduced-serum stress conditions. Neuro Oncol 5: 244-254.

7. Kunapuli P, Lo K, Hawthorn L, Cowell JK (2010) Reexpression of LGI1 in glioma cells results in dysregulation of genes implicated in the canonical axon guidance pathway. Genomics 95: 93-100.

8. Takahashi S, Yamada-Okabe H, Hamada K, Ohta S, Kawase T, et al. (2011) Downregulation of uPARAP mediates cytoskeletal rearrangements and decreases invasion and migration properties in glioma cells. J Neurooncol 103: 267-276.

9. Le PU, Angers-Loustau A, de Oliveira RM, Ajlan A, Brassard CL, et al. (2010) DRR drives brain cancer invasion by regulating cytoskeletal-focal adhesion dynamics. Oncogene 29: 4636-4647.

10. Cheung HC, Hai T, Zhu W, Baggerly KA, Tsavachidis S, et al. (2009) Splicing factors PTBP1 and PTBP2 promote proliferation and migration of glioma cell lines. Brain 132: 2277-2288.

11. Chow LM, Endersby R, Zhu X, Rankin S, Qu C, et al. (2011) Cooperativity within and among Pten, p53, and Rb pathways induces high-grade astrocytoma in adult brain. Cancer Cell 19: 305-316.

12. Bardot V, Dutrillaux AM, Delattre JY, Vega F, Poisson M, et al. (1994) Purine and pyrimidine metabolism in human gliomas: relation to chromosomal aberrations. Br J Cancer 70: 212-218.
